# Xiaoyao-Qingluoyin Cure Adjuvant-Induced Arthritis by Easing LPS Response-Related Pathway-Mediated Immune Abnormality

**DOI:** 10.1155/2022/8536998

**Published:** 2022-04-25

**Authors:** Dan-Feng Li, Chen-Qiong Xie, Yi-Jin Wu, Chao Shi, Chao-fan Ji, Yi-Fang Hu, Qiang Liao, Yan Li

**Affiliations:** ^1^Department of Traditional Chinese Medicine, The First Affiliated Hospital of Wannan Medical College (Yijishan Hospital), Wuhu, China; ^2^Research Center of Xin'an Medicine, Wannan Medical College, Wuhu, China; ^3^Department of Pharmacy, The Third Affiliated Hospital of Zhejiang Chinese Medical University, Hangzhou, China; ^4^Department of Pharmacy, The Second Affiliated Hospital of Wannan Medical College, Wuhu, China

## Abstract

Qingluoyin (QLY) is a representative herbal formula prescribed for hot symptom-related rheumatoid arthritis treatment. Among its derivatives, Xiaoyao-Qingluoyin (XYQLY) attracts increasing attention due to the notable clinical efficacy. In this study, we compared its effects with QLY on adjuvant-induced arthritis (AIA) in rats and partially elucidated the antirheumatic mechanism using a network pharmacology-based strategy. After continuous oral treatments, clinical outcomes were systematically evaluated by radiographic, histological, immunohistochemical, and serological analyses. Possibly altered pathways were predicted based on reported interactions between the related chemicals and proteins/genes. The obtained conclusion was further validated by experiments *in vitro*. QLY and XYQLY eased polyarthritis in AIA rats after repeated doses, which reflected in reduced inflammation and bone degradation and downregulated p-p65, MMP3, and TLR4 expressions in joints. Meanwhile, they restored oxidative stress (MDA, SOD, GSH, T-AOC, and NO) and inflammatory indicators (TNF-*α* and CO) in serum. Synovium-based immunoblotting assay revealed that QLY and XYQLY were similarly effective in downregulating MMP3 and COX-2, but XYQLY treatment exhibited notable merit in suppressing p-p65 expression. Network pharmacology analysis hinted that XYQLY should exert greater impacts on LPS signaling and the downstream. Based on results from LC-MS analysis, we treated AIA rat-derived peripheral blood mononuclear cells with either QLY or XYQLY-based chemical combinations and confirmed that XYQLY had the better potential in inhibiting TLR4/NF-*κ*B-controlled IL-6 production. Consequently, it led to a more profound decrease in Th17 cells counts. Overall evidence demonstrated that XYQLY was especially effective in regulating innate immunity and, therefore, improved immune environment in AIA rats as a whole.

## 1. Introduction

Rheumatoid arthritis (RA) is the most common autoimmune disease, which affects almost 1% of the total population worldwide. Without effective medication, it will gradually limit the motor functions of joints and eventually lead to disabilities. Hence, it imposes significantly negative effects on life quality of the patients and brings a heavy burden on both family and society [1, 2]. Furthermore, it is always accompanied by many extra-articular manifestations. The premature death caused by RA complication cardiovascular diseases further aggravates anxiety [3]. To deal with this public health issue, many endeavors have been made, and there are many available antirheumatic drugs nowadays. Unfortunately, even the most successful conventional disease-modifying antirheumatic drugs (cDMARDs) cannot always achieve the anticipated clinical outcomes, and the therapeutic efficacy is hard to be sustained [4]. Despite this, the need for these drugs is continuously increasing. It is estimated that their marketing volume is only smaller than antineoplastic and far exceeds many other medicines [5]. Under these backgrounds, developing new antirheumatic reagents is not only clinically necessary but also very lucrative. This work is challenging, and many factors including clinical performance, safety profile, and economic affordability should be simultaneously taken into consideration [1, 6].

Thanks to the accumulating knowledge about the etiology of RA, it is now possible for us to disrupt some key pathological events and thereby improve the prognosis. It paves the way for introducing the novel biological DMARDs into clinical practices [1, 7]. However, these regimens are expensive, and their safety concerns have not been thoroughly addressed. Besides, before the next breakthrough in understanding RA pathology, we cannot expect a great leap forward in the clinical efficacy improvement. Comparatively, traditional medicines could be a good alternative to resolve the dilemma. Almost all the continuously surviving civilizations have their own traditional medicine systems. There are many valuable records of successful RA treatment strategies in traditional Chinese medicine (TCM) archives. Qingluoyin (QLY) is one of the effective TCM antirheumatic formulas validated by both clinical and experimental evidence [8, 9]. It was prescribed by a famous contemporary TCM master Jiren Li and specifically used in treating hot symptom-related RA (active RA). Besides promising therapeutic effects, QLY also has obvious safety merits. Similar to other herbal formulas, its chemical composition is complicated. As a result, QLY simultaneously targets multiple targets. This characteristic is not only essential for the therapy of a systematic disease like RA but also amplifies the difficulty in elucidating the therapeutic mechanisms. Therefore, although some progresses have been achieved, relevant research studies are still ongoing.

Meanwhile, to further improve the clinical performance, many QLY derivatives are designed and introduced into clinical practice. Among them, Xiaoyao-Qingluoyin (XYQLY) attracts increasing attention due to its notable clinical efficacy. It is a hybrid of QLY and Xiaoyaosan (XYS), a famous herbal formula documented in an ancient Chinese pharmacopeia Prescriptions of People's Welfare Pharmacy. According to TCM theory, the change in formulation will strengthen its capability in expelling pathogenic heat and dampness and thereby be more effective in treating RA-related inflammatory manifestation. Preliminary clinical observation basically confirmed this speculation. However, we still lack solid evidence to further support this claim. In this study, we compared the therapeutic efficacy of QLY and XYQLY on adjuvant-induced arthritis (AIA) in rats and attempted to explain the possible advantages of XYQLY by using a network pharmacology-guided mechanism research.

## 2. Materials and Methods

### 2.1. Chemicals and Reagents

Incomplete Freund's adjuvant (IFA) and *Bacillus* Calmette-Guérin (BCG) were purchased from Rebio Scientiﬁc (Shanghai, China). TNF-*α*, IL-1*β*, IL-10, and IL-6 ELISA kits were the products of Multi Science (Hangzhou, China). Biochemical quantification kits for the determination of malondialdehyde (MDA), reduced glutathione (GSH), total superoxide dismutase activity (SOD), total antioxidant capacity (T-AOC), nitric oxide (NO), nitric oxide synthase (inducer) (iNOS), and carbonic oxide (CO) were brought from Jiancheng Bioengineering Institute (Jiangsu, China). The primary antibodies used in immunoblotting and immunohistochemical experiments including anti-MMP3, COX-2, TLR4, SIRT1, PPAR-*γ*, p65, p-p65, and *β*-actin antibodies together with secondary antibodies were provided by ABclonal Technology (Wuhan, China). Fluorescein-tagged anti-CD3, CD4, and IL-17*α* antibodies for the use of flow cytometry analysis were supplied by Beyotime Biotech (Nantong, Jiangsu, China). Rat peripheral blood mononuclear cell (PBMC) isolation kit, TaqMan RT-qPCR kit, and cDNA synthesis kit were purchased from Solarbio Technologies (Beijing, China). All the solvents were of analytical grade and supplied by Merck Chemicals (Shanghai, China). High-purity compounds (>98%) kaempferol, paeoniflorin, matrine, sophocarpine, and sinomenine were all obtained from Yuanye Bio-Technology (Shanghai, China).

### 2.2. Preparation of Herbal Extracts

Four components of QLY including Radix Sophorae Flavescentis (Kushen, KS), Caulis Sinomenii (Qingfengteng, QFT), Phellodendri Cortex (Huangbo, HB), and Rhizoma Dioscoreae Tokoro (Bixie, BX) together with the other ingredients included in XYQLY namely Radix Bupleuri (Chaihu, CH), Radix Angelica Sinensis (Danggui, DG), Radix Paeoniae Alba (Baishao, BS), and Rhizoma Atractylodis Macrocephalae (Baizhu, BZ) were all purchased from Tongrentang Co., Ltd. (Bozhou, Anhui, China) and authenticated by associated professor Jian Zuo (Wannan Medical College, Wuhu, Anhui, China). The voucher specimens (ID: QLY-2020-007-013/028) were deposited in the Herbarium Center, Wannan Medical College, China. The raw herbal materials were soaked in water for 1 h and then boiled for 30 min. This extraction procedure was repeated for 3 times. The combined decoction was filtrated and condensed into a sticky extract with the density of 1.2 g/ml with the aid of a rotary evaporator. Before further use, all the products were preserved under 4°C in a refrigerator (less than 2 weeks).

### 2.3. Induction of AIA and Treatments

Thirty-six 7 weeks old male Sprague Dawley rats were used in this study, which were purchased from Qinglongshan Experimental Animal Company (Nanjing, Jiangsu, China). All the animals were housed in a temperature-controlled room (24 ± 1°C) with a 12-hour light/dark cycle. They were allowed free access to food and water. After a quarantine period of 7 days, the rats were randomly divided into 4 groups: normal control group, AIA model control group, XYQLY treatment group, and QLY treatment group. To induce AIA, all the rats except for normal controls were subcutaneously injected with 0.1 ml freshly prepared complete Freund's adjuvant (CFA, comprised of IFA and heating-inactivated BCG, 15 mg/ml) in the right hind paw [10]. It was recorded as day 0. Since the next day, the animals were orally administered by either QLY or XYQLY for a period of 36 days. The total equivalent raw drug doses for them were 9.87 and 14.15 g/kg/day (divided into 3 aliquots), respectively. The normal and model controls were treated with normal saline instead. The animal experimental protocol was approved by the Ethics Committee of Wannan Medical College, Anhui, China, and all the experimental procedures were strictly in accordance with the Guidelines for the Use and Care of Animals, Wannan Medical College.

### 2.4. Assessment of Arthritic Severity

Since CFA immunization, the arthritic score was periodically assessed by 3 scholars, who were blinded to the experimental arrangement. Quantification criterion of the scale 0–4 was defined as following [10]: 0, no signs of morphological changes; 1, slight erythema; 2, significant redness and swelling; 3, severe local inflammatory manifestations; and 4, severe swelling and arthrodesis. Joint structures of the hind paws were preliminarily examined by digital radiographs (DRs) on day 35 under anesthesia. Before sacrifice, maximum amount of blood was collected. One portion of anticoagulation blood was immediately subject to complete blood cell count (CBC) using an automatic hematology analyzer (Prokan, Shenzhen, China). The remaining anticoagulation blood was used to separate PBMCs. Levels of MDA, SOD, GSH, TNF-*α*, and CO in serum were detected by corresponding kits according to the protocols. After rats were killed, the paws were dissected and fixed in 10% neutral formalin. After being decalcified with 10% EDTA for 1 month, the specimens were embedded in paraffin, sliced to 3 *μ*m thickness, and finally stained with hematoxylin and eosin (H & E). The pathological changes within joints were observed under a light microscope (Olympus BH-2, Tokyo, Japan).

### 2.5. Evaluation of mRNA/Protein Expression

In immunohistochemical experiments, the dewaxed slices were treated with H_2_O_2_ and citric acid in turn to inactivate the endogenous peroxidase and repair the antigen. Subsequently, the specimens were incubated with normal serum, primary antibodies, and HRP-conjugated secondary antibody step by step. The immunized proteins were visualized after further incubation with a DAB substrate mixture, and hematoxylin-based counterstaining was finally performed.

Whole proteins in fresh synovial tissues from knee joints were extracted using RIPA buffer supplemented with PMSF and protease inhibitors by the means of interval ultrasound treatments. Subsequently, the samples spiked into dilution buffer were denatured by boiling. Samples with quantified proteins were then separated by SDS-PAGE and transferred to PVDF blotting membranes. Thereafter, the membranes were blocked with 5% BSA and incubated with specific primary antibodies at 4°C overnight, which was followed by further incubation with appropriate secondary antibodies at room temperature for 1 h. Finally, the signals were developed using an ECL detection kit on a Tanon 5200 system (BioTanon, Shanghai, China).

Total RNA in some other fresh synovial tissues was extracted by TRIzol reagent. After chloroform extraction, the organic phase was discarded. RNA within the aqueous phase was precipitated by isopropanol and further purified with 75% ethanol. Using a reverse transcription kit provided by Applied Biosystems (Foster City, CA, USA), the samples were synthesized into cDNA. The resulting products were subsequently subjected to qPCR procedure on a 7500 Real-Time PCR system (Thermo Fisher Scientific, Rockford, IL, USA). The 20 *μ*l reaction system was comprised of 1 *μ*l cDNA, 0.5 *μ*l forward primer, 0.5 *μ*l reverse primer, 10 *μ*l qPCR reaction mixture, and 8 *μ*l DEPC water. Sequences of the primers were detailed as follows: *β*-actin, forward, TGTCCACCTTCCAGCAGATGT, reverse, AGCTCAGTAACAGTCCGCCTAGA; iNOS, forward, TGCCTTTGCTCATGACATCG, reverse, AACACGTTCTTGGCGTGGA; and IL-1*β*, forward, TCCTCTGTGACTCGTGGGAT, reverse, TCAGACAGCACGAGGCATTT. Relative expression of mRNA was calculated based on the 2-ΔΔCt method by taking *β*-actin as the internal reference.

### 2.6. In Vitro Treatments of PBMCs

Rat PBMCs were maintained in RPMI 1640 medium supplemented with 10% FBS immediately after isolation. The cell culture was performed in a humidified atmosphere with 5% CO_2_ at 37°C. All the cells were directly used without any further passages. AIA rat-derived PBMCs were stimulated with various chemical combinations, and some other normal PBMCs and untreated AIA PBMCs were taken as controls. Twelve hours later, both the cells and medium are collected. Levels of IL-1*β*, IL-6, and IL-10 in the medium were determined using ELISA kits in accordance with the manufacturer's instructions. Expressions of proteins TLR4 and p-p65 and expressions of mRNA IL-1*β* and iNOS in the cells were evaluated by immunoblotting and RT-qPCR methods, respectively. The experimental procedures were detailed as above. Distribution of Th17 cells in these PBMCs was analyzed by the means of flow cytometry, and this subset was identified as CD3^+^CD4^+^CD17*α*^+^ lymphocytes.

### 2.7. Network Pharmacology Analysis

The prediction of possible therapeutic targets was achieved by using data documented in TCMSP (traditional Chinese medicine systems pharmacology, https://tcmspw.com/tcmsp.php). Chemicals from the formulas were first screened using the following criterion: oral relative bioavailability (OB) ≥30% and drug-likeness (DL) ≥0.18. Reported targets related to the kept compounds were obtained from the same public library and enriched in Gene Ontology (GO) pathways. The results (*p* < 0.05) were subsequently ranked and visualized by an online tool Metascape (https://metascape.org/). Detailed information about the possibly altered pathways was retrieved from the database DisGeNET (https://www.disgenet.org/) or GeneCards (https://www.genecards.org/). The network diagram exhibiting interactions between the bioactive components and a certain pathway was constructed by Cytoscape 3.7.0.

### 2.8. LC-MS Analysis

The sufficiently diluted XYQLY and QLY extracts were centrifuged at 12,000 rpm for 15 min, and 300 *μ*l supernatant obtained was then spiked into 1,000 *μ*l extract solution (methanol : water = 4 : :1). After 30 s vortex, the mixture was filtered through a 0.22 *μ*m filter membrane and directly fed to LC-MS instrument (a Thermo Scientific Horizon UHPLC System coupled with a *Q* Exactive Focus mass spectrometer) for analysis. The chromatographic separation was achieved on a Waters UPLC BEH C18 column (1.7 *μ*m 2.1^∗^100 mm). The flow rate and injection volume were set at 0.4 ml/min and 5 *μ*l, respectively. The solvent 0.1% formic acid in water and 0.1% formic acid in acetonitrile served as phase A and phase B, respectively. The multistep gradient elution program was described as below: 0–3.5 min, 95–85% A; 3.5–6 min, 85–70% A; 6–6.5 min, 70–70% A; 6.5–12 min, 70–30% A; 12–12.5 min, 30–30% A; 12.5–18 min, 30-0% A; 18–25 min, 0–0% A; 25–26 min, 0–95% A; and 26–30 min, 95–95% A. Main parameters for the mass spectrometer were summarized as follows: sheath gas flow rate, 45 Arb; aux gas flow rate, 15 Arb; capillary temperature, 400°C; full ms resolution, 70000; MS/MS resolution, 17500; collision energy, 15/30/45 in NCE mode; and spray voltage, 4.0 kV (positive) or −3.6 kV (negative). The data were acquired and processed by Xcalibur software.

### 2.9. Statistical Analysis

All the data acquired were recorded as mean ± standard deviation. The statistical analyses were performed with the aid of GraphPad Prism 8.0 Software (Cary, NC, USA). When *p* value <0.05 or 0.01, the difference was taken as statistically significant.

## 3. Results

### 3.1. Both QLY and XYQLY Significantly Alleviated AIA

**Figure 1:**
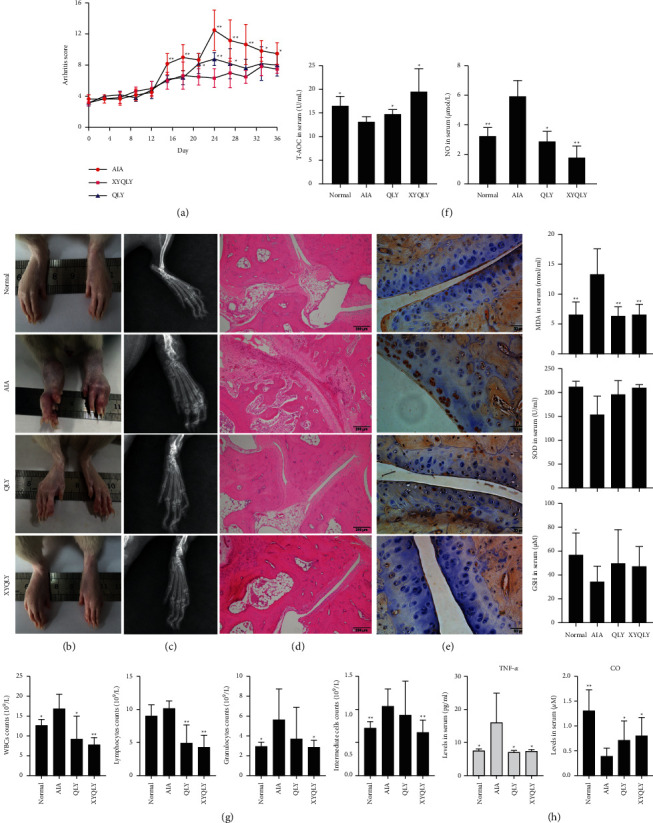
Therapeutic effects of QLY and XYQLY treatments on AIA rats. (a) Periodic arthritic score changes; (b) morphological observation of hind paws (yellow arrow, local swelling and edema); (c) DR examination of left hind paw (yellow arrow, joint cavity narrowing); (d) histological examination of interphalangeal joints of left hind paw (yellow arrow, cartilage erosion); (e) local expression of MMP3 in cartilage (investigated by immunohistochemical method; yellow arrow, chondrocytes highly expressing MMP3); (f) levels of SOD, GSH, MDA, T-AOC, and NO in serum; (g) results of CBC; and (h) levels of TNF-*α* and CO in serum. Statistical significance: ^*∗*^*p* < 0.05 and ^*∗∗*^*p* < 0.01 compared with AIA models.

As shown in Figure 1(a), both QLY and XYQLY reduced arthritis score a lot in the treated AIA rats since day 20. CFA-caused acute inflammation reached the peak on day 24, when XYQLY exhibited obvious advantages over QLY concerning their effects on arthritis score. By the end of the observational period, the differences in this index among groups were narrowed due to the spontaneously eased polyarthritis, but certain pathological changes can be still observed. Hind paws of AIA rats suffered from severe inflammation, which was not obvious in QLY- and XYQLY-treated rats. Comparatively, the arthritic condition of QLY-treated rats was worse than XYQLY-treated counterparts, as joints' deformation and bulbous inflammation can be still noticed in their paws (Figure 1(b)). DR examination confirmed the above findings. Similar to healthy rats, joints' structure of XYQLY-treated rats was intact. Significant joints' cavity narrowing and bone density loss occurred in AIA models. These situations were greatly improved after QLY treatment (Figure 1(c)). In the histological examination, we found extensive inflammatory infiltration and cartilage degradation in interphalangeal joints of AIA rats, while both the treatments prevented these pathological changes (Figure 1(d)). Their protective effects on joints were further validated by MMP3 changes. The increased MMP3 expression in cartilage in AIA rats was abrogated by both QLY and XYQLY therapies (Figure 1(e)). Meanwhile, the treatments restored levels of GSH and SOD. Of note, they effectively reduced serological MDA, T-AOC, and NO levels in the treated rats (Figure 1(f)). CBC further confirmed the anti-inflammatory potentials of these formulas. Major types of white blood cells (WBCs) including lymphocytes, granulocytes, and intermediate cells in peripheral blood of AIA rats were decreased after treatments. Comparatively, XYQLY-caused decrease was more profound (Figure 1(g)). Production of TNF-*α* was similarly inhibited by QLY and XYQLY, while a novel anti-inflammatory mediator CO was increased by them (Figure 1(h)).

### 3.2. XYQLY Was More Effective than QLY in Controlling AIA-Related Inflammation

The XYQLY exhibited some therapeutic advantages than QLY based on the above observations.

**Figure 2:**
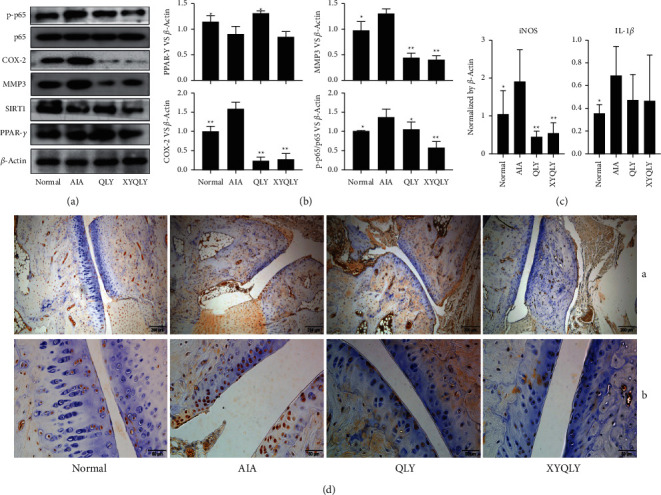
The effects of treatments on the inflammatory environment in joints of AIA rats. (a) Expressions of proteins PPAR-*γ*, SIRT1, MMP3, COX-2, p65, and p-p65 in the synovium, investigated by immunoblotting method; (b) quantification results of assay A; (c), expression of mRNA iNOS and IL-1*β* in the synovium, investigated by RT-qPCR method; and (d) local expressions of p-p65 (A) and TLR4 (B) in the synovium (investigated by immunohistochemical method; red arrow, cells highly expressing either p-p65 or TLR4). Statistical significance: ^*∗*^*p* < 0.05 and ^*∗∗*^*p* < 0.01 compared with AIA models.

However, we cannot be certain that XYQLY has better anti-inflammatory potential than QLY, because they were similarly effective in regulating oxidative stress and inflammatory indicators. This discrepancy could be caused by the spontaneous remission of systematic inflammation during the later stage of AIA, especially in tissues unrelated to arthritis. Therefore, we subsequently focused on changes in flamed paws. It was found that QLY and XYQLY can both be substantially downregulated MMP3 in the synovium, which was consistent with results obtained from the immunohistochemical analysis. Also, they suppressed the expression of COX-2 to a similar extent (Figure 2(a)). Interestingly, XYQLY seemed to be more effective in inhibiting the NF-*κ*B pathway, as it caused more decrease in p-p65 expression than in QLY (Figure 2(b)). The previous investigation revealed that QLY cured experimental arthritis by disrupting energy metabolism-immune feedback. Hence, we investigated two key metabolic regulators SIRT1and PPAR-*γ*. To the disappointment, XYQLY did not show any merits in this regard (Figure 2(a)). The following PCR analysis demonstrated the anti-inflammatory potentials of the formulas once again. They downregulated mRNA IL-1*β* expression a bit, and more importantly, mRNA iNOS expression was significantly suppressed (Figure 2(c)). In the following immunohistochemical examination, we observed that XYQLY thoroughly scavenged the accumulated p-p65 in joints, exhibiting more powerful effects in inhibiting NF-*κ*B than QLY. But at the same time, we found that XYQLY did not further reduce TLR4 expression there, a key upstream of NF-*κ*B (Figure 2(d)). Nonetheless, these clues suggested that XYQLY can effectively ease inflammation by downregulating TLR4/NF-*κ*B.

### 3.3. Lipopolysaccharide (LPS) Signaling Was a Therapeutic Pathway of XYQLY

**Figure 3:**
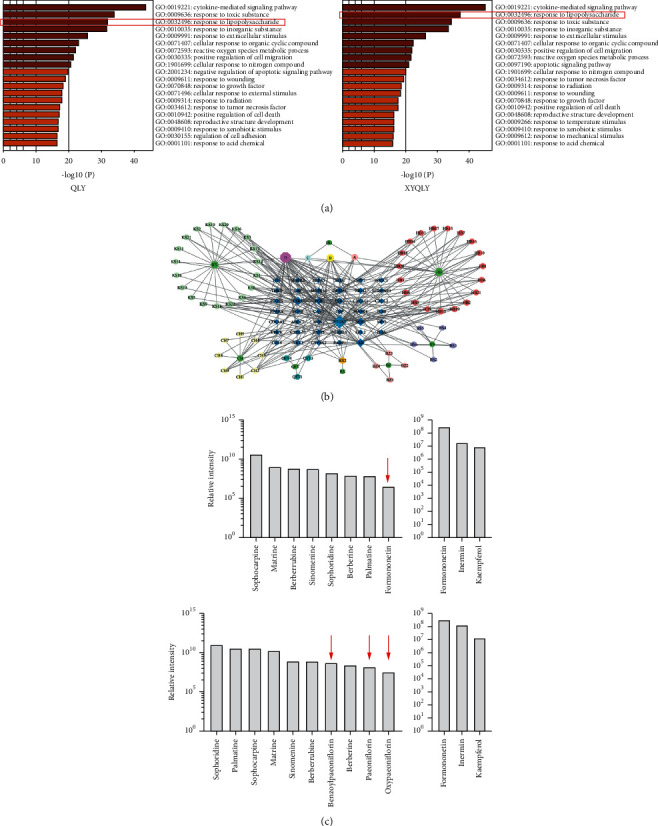
Identification of therapeutic targets and related chemicals of the formulas. (a) GO pathway enrichment analysis based on reported targets of chemicals derived from QLY and XYQLY; (b) the interactions among chemicals and LPS response-related genes; and (c) LPS signaling-related compounds within the formulas detected by LC-MS analysis. The 4 subfigures represent compounds closely related to the regulation of the LPS signaling pathway, namely, formononetin, benzoylpaeoniflorin, paeoniflorin, and paeoniflorin. Red arrows indicate compounds closely related to the regulation of LPS.

Using data from TCMSP, we considered possible targets for these two formulations. Biologically active compounds and corresponding targets are included in Supplement S1. Based on these targeted proteins/genes, we performed GO pathway enrichment, and the results are shown in Figure 3(a). Among the top-ranking pathways, LPS response is especially notable, not only because it is indispensable for innate immunity but also because its hyperactivation is deeply implicated in RA-related inflammation [11]. By the modification of formulation, its importance is even further increased in XYQLY-based therapy, situated only after cytokine-mediated pathways. Hence, we displayed the interaction between bioactive components and LPS signaling in Figure 3(b). It can be observed that KS and HB mostly contribute to the inhibitory effects of QLY on LPS-related responses. The supplement of CH, BZ, and BS in XYQLY further reinforced effects on this pathway. All abbreviations of the compounds were defined in Supplementary S1. Subsequently, we sought to clarify whether these compounds were present in the decoctions of these formulations. Total ion chromatograms of LC-MS analysis were displayed in Supplementary S2. The compounds identified in QLY and XYQLY were listed in Supplementary S3 and Supplementary S4, respectively. In Figure 3(c), we selectively displayed the compounds with close relevance to the regulation of LPS signaling. All the bioactive components from QLY can be detected in XYQLY. Compared with DG, CH, and BZ, BS brought more profound changes in the chemical composition of XYQLY. All the extra compounds identified in this formula were derived from this raw drug in this analysis, including benzoylpaeoniflorin, paeoniflorin, and oxypaeoniflorin. Due to the increased counts of LPS pathway-targeting compounds, it supported the notion that XYQLY was more effective in controlling innate immune-related acute inflammation.

### 3.4. XYQLY Improved Inflammatory Environment by Suppressing TLR4/NF-*κ*B

**Figure 4:**
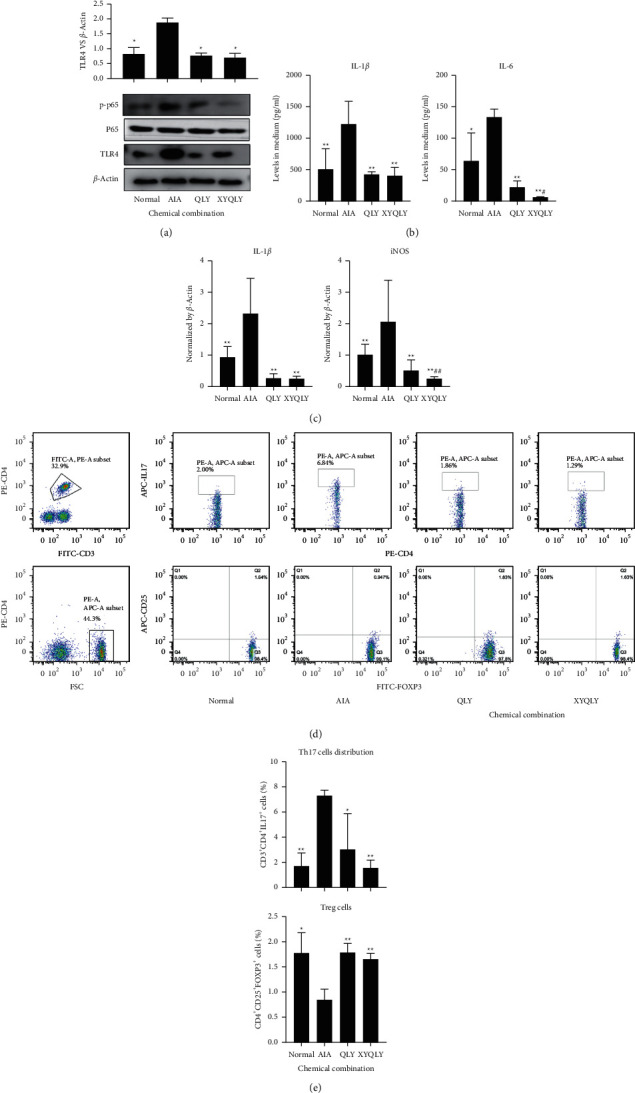
The effects of different chemical combinations on in vitro cultured AIA PBMCs. (a) Expressions of proteins TLR4 and p-p65 in PBMCs, investigated by immunoblotting method; (b) levels of IL-1*β* and IL-6 in cell culture medium; (c) expressions of mRNA iNOS and IL-1*β* in PBMCs, investigated by RT-qPCR method; (d) flow cytometry analysis of Th17 cells in PBMCs; and (e) quantification results of assay D. Statistical significance: ^*∗*^*p* < 0.05 and ^*∗∗*^*p* < 0.01 compared with AIA PBMCs; ^#^*p* < 0.05 and ^##^*p* < 0.01 compared with QLY chemical combination-treated AIA PBMCs.

**Figure 5:**
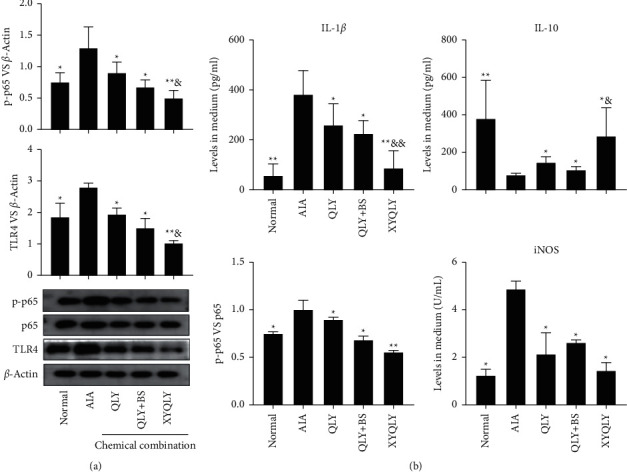
The effects of different chemical combinations on in vitro cultured AIA PBMCs. (a) Expressions of proteins TLR4 and p-p65 in PBMCs, investigated by immunoblotting method; (b) levels of IL-1*β*, IL-10, and iNOS in cell culture medium. Statistical significance: ^*∗*^*p* < 0.05 and ^*∗∗*^*p* < 0.01 compared with AIA PBMCs; ^&^*p* < 0.05 and ^&&^*p* < 0.01 compared with QLY + BS chemical combination-treated AIA PBMCs.

Based on their abundance in decoction and importance to LPS signaling regulation, matrine, sophocarpine, sinomenine, and kaempferol were chosen as representative bioactive compounds of QLY and used to form a chemical combination in the following experiments. According to their relative abundance in QLY and reported effective concentrations, their treatment concentrations were set at 2, 8, 2, and 6 *μ*g/ml [12, 13]. To simulate the chemical profile of XYQLY, 4 *μ*g/ml paeoniflorin was added. TLR4/NF-*κ*B is the most important downstream of LPS signaling, and we, therefore, investigated the effects of the two chemical combinations on this pathway in AIA PBMCs. It is obvious that the decrease in p-p65 expression under XYQLY stimulus was more significant than the QLY chemical combination, which confirmed the conclusion from network pharmacology analysis (Figure 4(a)). ELISA analysis revealed that although XYQLY treatment caused a similar decrease in IL-1*β* release to QLY, it led to a more significant decrease in IL-6 production in these cells (Figure 4(b)). PCR analysis found XYQLY- and QLY-derived chemicals were similarly effective in reducing IL-1*β* expression in AIA PBMCs, but their capability in controlling macrophage M1 polarization is different. XYQLY more efficiently suppressed iNOS expression (Figure 4(c)). As IL-6 bridges innate and adaptive immunity, we investigated the effects of these treatments on the differentiation of Th17 cells in AIA PBMCs (Figure 4(d)). As anticipated, compared with normal samples, an enlarged Th17 cell population was found in AIA PBMCs, and both chemical combinations efficiently reduced their counts. It further showed the advantages of XYQLY in regulating immune status. More decrease in Th17 cells was achieved by XYQLY than QLY (Figure 4(e)). In addition, we found that BS is the main core drug of XYQLY, so is it possible to achieve the same curative effect as XYQLY only by adding total glucosides of paeony? So we did further validation in vitro. Obviously, the expressions of p-p65 and TLR4 more significantly decreased under XYQLY stimulation than the chemical combination of QLY + BS (Figure 5(a)). ELISA analysis revealed that although XYQLY treatment caused a decrease in IL-1*β* and iNOS release similar to QLY, XYQLY treatment caused a more significant decrease in IL-1*β* and iNOS release than QLY + BS (Figure 5(b)). Interestingly, XYQLY restored the expression of IL-10 in PBMCs, which was significantly higher than QLY + BS. In conclusion, the XYQLY compound has a more obvious downregulation effect on TLR4/NF-*κ*B pathway in AIA PMCS than QLY + BS.

## 4. Discussion

The QLY is defined as a cold-natured TCM formula. It embodies a basic therapeutic TCM strategy against RA. Most active RA-related symptoms including pain, inflammation, and accelerated energy metabolism can be attributed to pathogenic heat. Thus, a priority of TCM antirheumatic treatments is to expel this evil. Available evidence confirmed that QLY can reduce inflammatory cytokines in both RA patients and rats with experimental arthritis and systematically eased inflammation [14–16]. Meanwhile, it reshapes energy metabolism in arthritic subjects, which is favorable for the sustained remission of inflammation. In addition, it has promising potential against synovial angiogenesis and, therefore, can slow down the progress of synovitis [17, 18]. However, it should be noticed that RA is *s* heterogeneous disease according to both TCM and Western medicine theories. Although most RA patients share some common pathological characteristics, their differences cannot be overlooked. Thus, personalized medication is totally necessary. In fact, treatment based on syndrome differentiation is a common clinical practice in TCM. Taking RA as an example, besides the basic conception of cold and hot subtypes, many other clinically meaningful clues should be taken into consideration too. First, women are more susceptible to this disease [1–3]. Second, depression incidence is higher in RA patients than the general population [19]. Third, blood stasis is a common secondary factor for arthritic changes. Because of these, the combination of QLY and XYS is reasonable. As one of the most famous TCM formula, XYS has been used in China for nearly 1000 years. It was originally designed to nourish blood, strengthen spleen functions, and relieve liver-related depression. Numerous evidence has confirmed its excellent capability in treating psychological and gynecological diseases [20, 21]. As a main component of XYS, BS is a typical stasis-eliminating drug. The above facts hint that the combined use of XYS with antirheumatic medicines may be beneficial for RA treatments, which was validated by many experiments [22]. Under these contexts, it is not surprising to notice that XYQLY performs better than QLY.

Initially, we thought the effects of XYQLY on the nerve system contributed a lot to the improved antirheumatic efficacy, considering its possible influence on depression and the newly conceptualized cholinergic anti-inflammatory pathway (CAP). Unfortunately, the preliminary assays found that neither acetylcholine nor *α*7nAChR (two key components in CAP) was substantially affected by XYQLY during the treatment. Results from the current network pharmacology analysis are not very novel but helpful for explaining the clinical observations. LPS response-related pathway is essential for the innate immune system to fulfill its defensive functions [23]. But its hyperactivation always causes excessive inflammation and is deeply implicated in inflammatory diseases. Nowadays, TLR4-based LPS signaling has been recognized as an effective therapeutic target for the treatment of RA [24]. By downregulating its key downstream targets such as NF-*κ*B, certain antirheumatic therapies can cripple the development of inflammatory monocytes/macrophages and consequently hinder the maturation of adaptive immunity [25]. Both LC-MS and in vivo pharmacological experiments confirmed that the supplement of BS is a key modification in the formulation of XYQLY, which eventually amplified the effects of QLY on LPS signaling. Total glucosides of paeony (TGP), the main bioactive components from BS, have been utilized as a successful antirheumatic reagent for decades [26]. Although the mechanism underlying its therapeutic actions on RA has not been thoroughly understood, convincing evidence shows that regulation of LPS signaling is involved in this regimen. Paeoniflorin, a representative compound from BS, can inhibit LPS-related responses in many different diseases models [27, 28]. Similarly, in this study, a small amount of paeoniflorin significantly amplified the effects of the QLY chemical combination on p-p65. It suggests that the targets of paeoniflorin and its derivatives are probably different from other chemicals from QLY, and the addition of BS will bring promising synergistic effects with other herbals on the regulation of LPS signaling. This deduction can be preliminarily witnessed in Figure 3(b). But the essence of traditional Chinese medicine lies in syndrome differentiation and treatment and holistic recuperation. As can be seen from Figure 5, XYQLY has a significantly higher impact on LPS signaling pathway than QLY adding BS compound.

Despite the inspiring findings, some issues need to be further elucidated. Arthritic score monitoring, morphological observation, and CBC all supported the better anti-inflammatory potentials of XYQLY than QLY (Figure 1). But confusingly, no significant difference was observed between XYQLY and QLY treatment groups concerning levels of TNF-*α*. A similar discrepancy was observed in vitro. XYQLY and QLY were similarly effective in reducing IL-1*β* production in AIA PBMCs, while XYQLY treatment caused a more obvious decrease in IL-6 levels (Figure 4). Based on available clues, we proposed a plausible theory that the conflicting results were affected by the stages of AIA. As well known, AIA-related inflammation cannot last for long. Although the synthesis of TNF-*α*, IL-6, and IL-1*β* is all mainly controlled by NF-*κ*B, their roles in inflammation are different. TNF-*α* and IL-1*β* are typically excreted by LPS-primed monocytes/macrophages via the activation of TLR4/NF-*κ*B. As AIA is induced by the exogenous pathogen BCG, its occurrence is largely mediated by the overwhelmed M1 monocytes/macrophages. Accordingly, the surge in TNF-*α* and IL-1*β* production is a hallmark of AIA-related inflammation during the early stages [29]. In this study, the samples were collected about 40 days after CFA immunization. At that moment, the effects of treatments on M1 monocyte/macrophage-released cytokines were narrowed due to the spontaneous remission of acute inflammation. On the other hand, IL-6 situates in the center of the inflammatory cascade and has the properties bridging innate and adaptive immunity [30]. Consistent with its pathological functions, its surge usually lags behind TNF-*α* and IL-1*β*, and certain lymphocytes also contribute to its production, besides from inflammatory monocytes/macrophages. Additionally, IL-6 is required for the development of many T cells implicated in RA, such as Th1 and Th17 cells [31]. Due to the significant decrease in IL-6, XYQLY effectively inhibited the differentiation of Th17 in PBMC of AIA rats. That is, XYQLY abrogated LPS response-related pathway in AIA rats and consequently improved the immune environment by reducing inflammatory cytokine production.

## Data Availability

The data used to support the findings of this study are available from the corresponding author upon request.

## Supplementary Materials

Supplementary Materials Supplementary S1. Abbreviations of the biologically active compounds, corresponding targets, and all compounds. Supplementary S2. LC-MS total ion chromatography analysis of QLY and XYQLY. Supplementary S3. The compounds identified in QLY. Supplementary S4. The compounds identified in XYQLY.
