# Supplementary material for: Xiaoyao-Qingluoyin Cure Adjuvant-Induced Arthritis by Easing LPS Response-Related Pathway-Mediated Immune Abnormality
Source: Evid Based Complement Alternat Med. 2022 Apr 25;2022:8536998. doi: 10.1155/2022/8536998 (PMC9060969; doi:10.1155/2022/8536998)
Supplement: Supplementary Materials — Supplementary S1. Abbreviations of the biologically active compounds, corresponding targets, and all compounds. Supplementary S2. LC-MS total ion chromatography analysis of QLY and XYQLY. Supplementary S3. The compounds identified in QLY. Supplementary S4. The compounds identified in XYQLY. [file 8536998.f1.pdf]

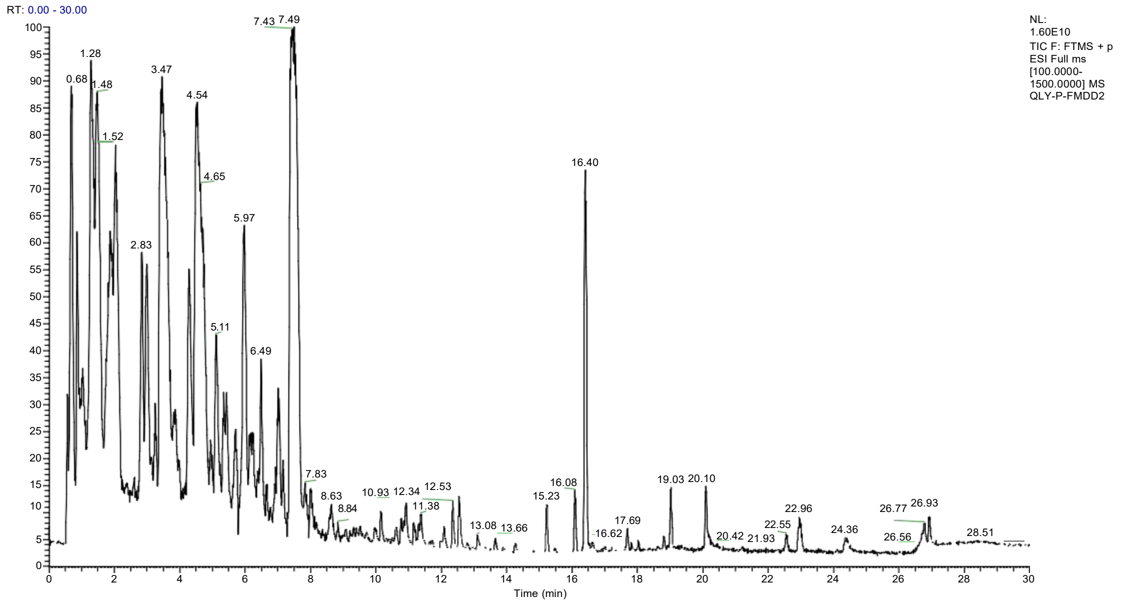

QLY-FMDD2 positive ion pattern TIC

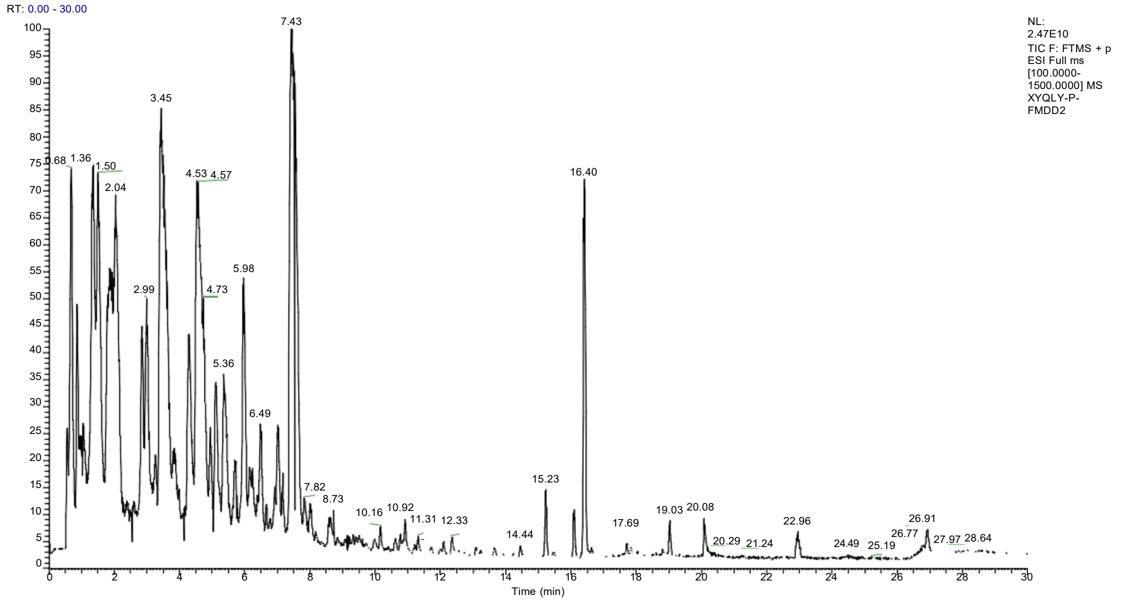

XYQLY-FMDD2 positive ion mode TIC

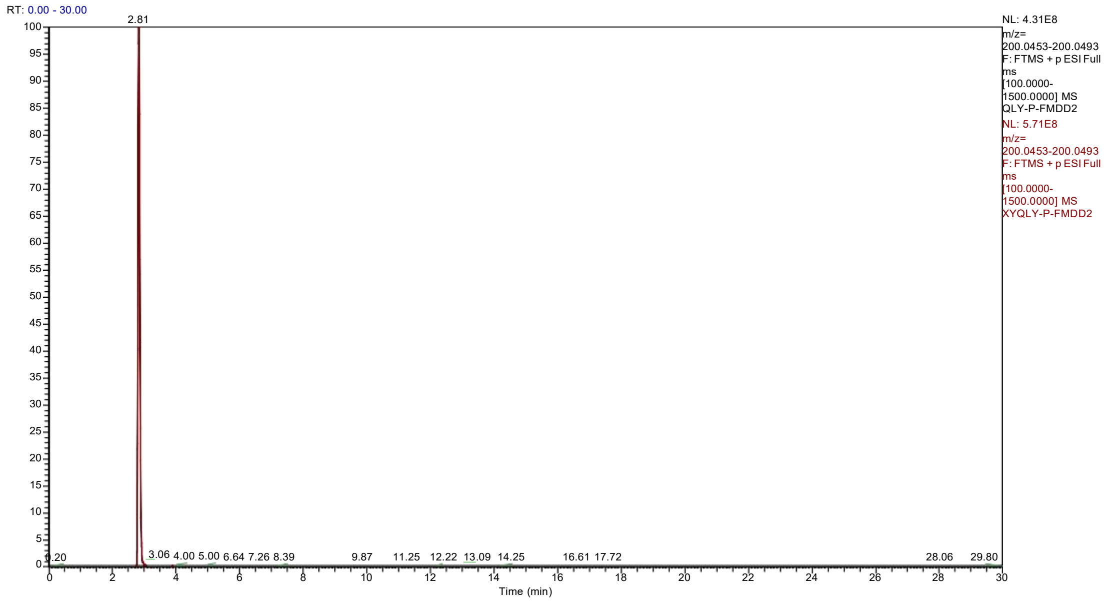

Internal standard overlap diagram of positive ion mode sample

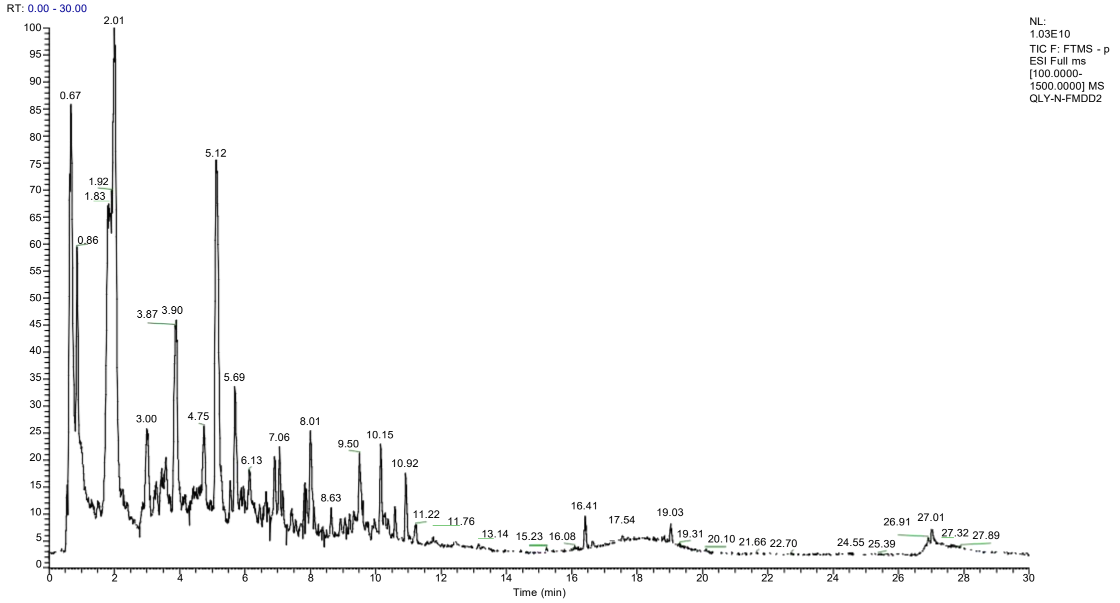

QLY-FMDD2 negative ion TIC mode

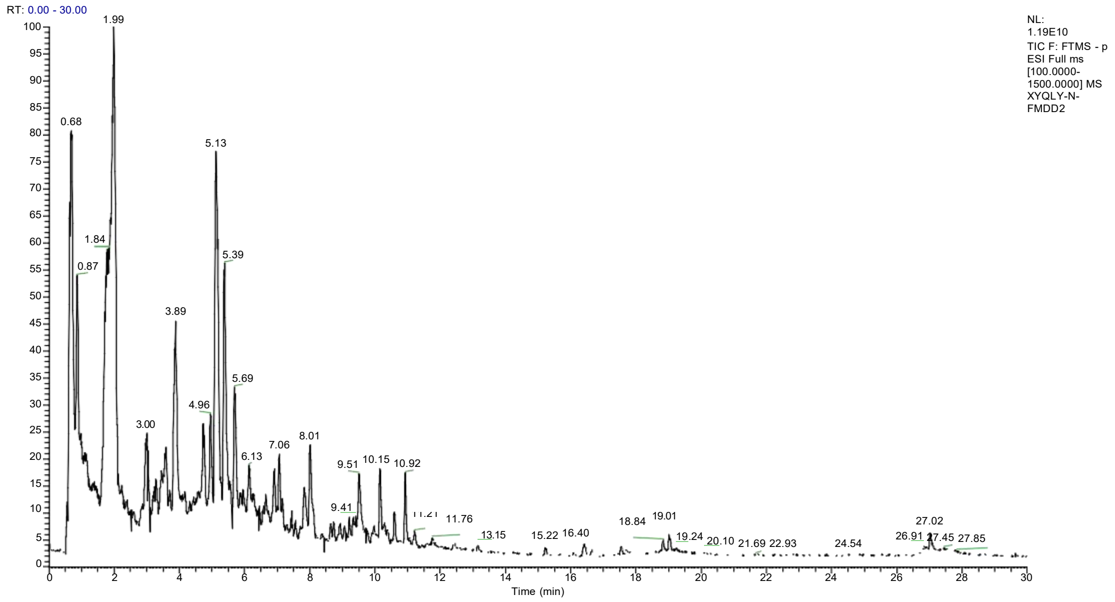

XYQLY-FMDD2 anion mode TIC

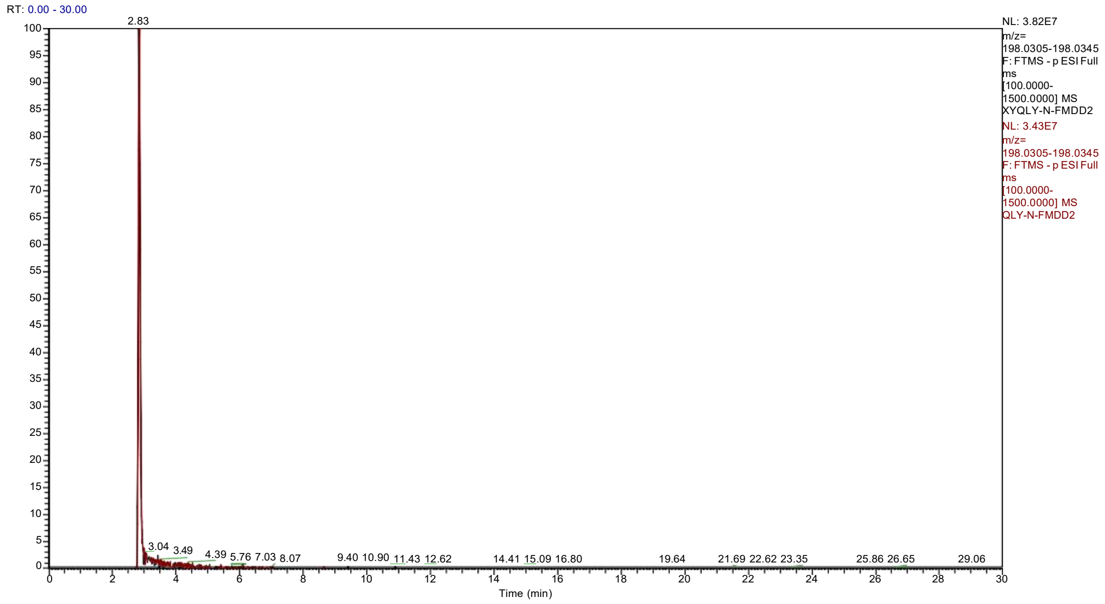

Anion mode internal standard overlap diagram
